# Suicide Rates and Antidepressant Prescribing: A Casual or Causal Relationship?

**DOI:** 10.1371/journal.pmed.0030220

**Published:** 2006-06-13

**Authors:** Bernhard Baune, Phillipa Hay

## Abstract

Baune and Hay discuss the strengths and limitations of an ecological study that found an inverse correlation between suicide rates and fluoxetine prescriptions.

In an ecological study in this issue of *PLoS Medicine*, Milane and colleagues found a temporal association between prescription of specific serotonin reuptake inhibitor (SSRI) antidepressants and suicide rates in the United States [ 1]. Their study addresses a subject of great public health importance and clinical interest. The researchers asked the question: was the use of antidepressants associated with a change in suicide rates in the general population of the United States between 1988 and 2002? There were two underlying hypotheses: (1) antidepressants can trigger suicide and subsequently increase suicide rates, and (2) through the treatment of depression with antidepressants, suicide rates decline over time.

## Methodology

To address these hypotheses, the researchers conducted an ecological study drawing on aggregated data on suicide in the US general population between 1960 and 2002. These data were collected from the annual statistical files of the National Vital Statistics System compiled by the US Centers for Disease Control. Data on fluoxetine prescription were obtained from IMS Health, a company that provides data on drug use to the pharmaceutical and health-care industries.

The authors estimated a potential effect of fluoxetine on suicide rates by the use of a time-series regression model. They assumed a predicted suicide mortality in 1988–2002 based on maintained pre-1988 suicide trends, and considering fluoxetine as a covariate for a potential effect on change of suicide rates between 1988 and 2002.

## Key Findings

The authors reported that suicide rates were most prominent in the following two age groups: 15–24 and 25–44 years of age (the upper age limit of the study population was 65 years of age). The authors found that suicide rates fluctuated between 12.2 and 13.7 per 100,000 people for the entire population from the early 1960s until 1988, but then the rates gradually declined (in men and women), with the lowest value of 10.4 per 100,000 people in the year 2000. They also found that the prescription of fluoxetine inversely increased in relation to the trend in suicide rates from 1988 to 2002. This inverse relationship was highly correlated (r _s_ = –0.92, *p* < 0.001).

There was an inverse correlation between suicide rates and fluoxetine prescriptions.

Milane and colleagues then modeled suicide rates in 1988–2002 based on pre-1988 suicide trends, and their modeling suggested that fluoxetine prescription decreases suicides, both in women and in men. Further modeling analysis showed that if pre-1988 trends (i.e., trends in the pre-SSRI era) were extrapolated through 2002, suicide numbers would have been higher by about 33,600 cases. The authors summarized their findings with the hypothesis that SSRIs may have saved 33,600 lives since their introduction.

## Strengths and Limitations of the Study

The main strength of this study is the authors' effort to address the crucial question of whether antidepressants do harm or good at a population level. Unfortunately, a research question such as this is not easy to answer.

As the authors acknowledge, suggestions that there may be a causal relationship between fluoxetine prescription and suicide rates would represent an overinterpretation of the results. In a study like this, it is also important to consider other potential explanations for the fall in suicide rates, such as improvements in the economy or improved management of depression by primary-care providers. Moreover, as the study did not include people above 65 years of age, who are known to have an increased risk of suicide (especially in men) compared with younger people, the findings are limited to adults up to 65 years of age.

Another limitation of this study was the use of fluoxetine as a model of SSRI use. Several effective SSRIs have been introduced since the arrival of fluoxetine, and these newer SSRIs may have had an additional potential impact on suicide rates. Finally, although the authors used the best available data on the number of prescriptions of fluoxetine, these estimations are not very accurate in terms of actual intake of antidepressants. As there are no reliable figures available on adherence to drug prescriptions at the population level, the real effect of antidepressants on suicide rates is difficult to estimate.

## Implications for Clinicians and Policymakers

Clinicians may be tempted to conclude from the study that the use of SSRIs leads to a decrease in depression and, subsequently, to a decrease of suicide attempts in their patients. Policymakers might prioritize the use of antidepressants at both primary and secondary health-care levels, assuming from the study that these drugs improve the lives of people with depression.

But an alternate view of the results is that they do not contribute substantial new knowledge and have no major clinical implications, since the effectiveness of antidepressants at improving depression is well established [ 2] (and there is some evidence of their potential to decrease suicide rates [ 3, 4]). Moreover, it is questionable if a cross-sectional ecological study has the potential to adequately address the authors' central research question of whether antidepressants do harm or good at a population level. In general, ecological and cross-sectional studies are suitable for generating new hypotheses and study questions, but not to answer analytic research questions as such.

## Conclusion

In our view, the single conclusion that can be drawn from this study is that there was an inverse correlation between suicide rates and fluoxetine prescriptions. Thus, the study does not support an association between increased suicide and increased fluoxetine prescription rates. This finding is of public health importance and should stimulate further scientific endeavors, in particular ecological research with a longitudinal design that considers additional factors such as economic data and a wider range of antidepressants with a potential impact on depression and suicide.

## Abbreviation

SSRI: serotonin reuptake inhibitor
